# Therapeutic ultrasound: myths and truths for non-portable in-clinic and portable home use ultrasound

**Published:** 2020-11-02

**Authors:** David Draper, Rajiv M Mallipudi

**Affiliations:** 1 Professor, Exercise Sciences, Brigham Young University, USA; 2 Unit Medical Director, Yale New Haven Health, Clinical Instructor, Yale School of Medicine, USA

Therapeutic ultrasound was first discovered prior to World War II when German scientists were experimenting with sonar in submarines and found an interesting biophysical effect. When sound was released from the vessel to detect objects in the surrounding water, the scientists noted that fish absorbed sound waves and died. Thus, the idea of ultrasound producing biological effects was born.^1^

Therapeutic ultrasound is one of the most commonly used modalities that clinicians employ to treat orthopedic and muscle injuries, and often used for pain relief.^2–4^ With appropriate use by a competent therapist, ultrasound can provide several benefits for treating strains, sprains, tissue healing and pain.^5–9^

However, therapeutic ultrasound is one of the most misunderstood and, therefore, one of the most misused and underused modalities. Unfortunately, the majority of the time that ultrasound is used, it is used by someone with little knowledge of the device or its use. At times, some healthcare professionals rarely read the literature concerning ultrasound. Instead they depend on being trained by their co-workers. If wrong parameters and inadequate dosimetry are used on the patient, there may be negligible or even adverse outcomes.^1^

The purpose of this article is two-fold. First, it aims to compare traditional ultrasound with wearable ultrasound. Second, this review provides corrections for mistakes clinicians often make when applying (and prescribing) ultrasound for treatment.

## Myth 1. Warm whirlpools, paraffin baths, and silicate-gel heating packs all produce therapeutic heat. Thus, there is no reason to employ ultrasound.

### Truth 1.

While these 3 modalities produce therapeutic heat, their depth of penetration is shallow.^10^ Several years ago, scientists compared ultrasound heat with whirlpools, paraffin bath and hot packs at 1cm depth in human triceps surae muscle. These 3 devices only raised the temperature 2°C at this therapeutic depth. At a greater depth (3cm), there was no temperature change for non-ultrasound-based techniques. At 1cm depth ultrasound raised the muscle temperature 6°C. At 3cm the muscle temperature was raised over 4°C. Therefore, when deep heat is desired, ultrasound is the modality of choice.^1^ Ultrasound generates deep heat by the absorption and conversion of ultrasound into thermal energy (rapid vibration of molecules) within the tissue matrix.

## Myth 2. Pulsed ultrasound does not heat tissues.

### Truth 2.

Research performed by Gallo^11^ shed light on the fact that pulsed ultrasound can heat treated tissues. Spatial Peak Intensity (SPI) is the maximum output over time. Spatial Average Temporal Peak (SATP) is the result of only pulsed ultrasound. The SATP during the ON time of a pulse is displayed as intensity on the machine’s output meter. Gallo compared muscle temperature increase during ultrasound application. In group 1, pulsed ultrasound at a 50% duty cycle was applied for 10 minutes at 1W/cm^2^ over a 2 ERA (effective radiating era). The second group received 10 minutes of continuous ultrasound at 0.5W/cm^2^. The results showed that both treatments applied modest heat to the target area. Based upon this, (SPI and SATP) should be included when reporting the parameters of therapeutic ultrasound.^11^

## Myth 3. Patients do not feel anything during an ultrasound treatment.

### Truth 3.

The only reason a patient may not feel therapeutic ultrasound in a device is when the power output is too low (low duty cycle or low intensity). For example, a treatment at a duty cycle of 25%, and an intensity of 0.5W/cm^2^, is too low to bring about a therapeutic benefit.^11^

## Myth 4. Increasing the ultrasound intensity (W/cm^2^) increases the depth of tissue penetration.

### Truth 4.

Both the intensity and frequency of the ultrasound determines the depth of penetration into the tissue for therapeutic treatment. Turning up the intensity will send a stronger ultrasonic signal into the tissue which will propagate deeper.^12^ The absorption rate of ultrasound is also proportional to frequency. The higher the frequency, the more quickly energy is absorbed. For example, with the ultrasonic intensity being held equivalent, 1MHz ultrasound would show a similar energy signature in tissue from 2.5–5cm deep, as 3MHz ultrasound would from the surface to 3cm deep.^13^

## Myth 5. Applying an ice pack prior to an ultrasound treatment increases tissue heating.

### Truth 5.

According to this theory, applying a cold pack to the tissues initiates physiological responses such as vasoconstriction and decreased blood flow. Thus, cooling the area not only results in decreased local temperature, but it may temporarily increase the density of the tissue being treated. This occurs by decreasing superficial attenuation and facilitating ultrasound transmission to deeper tissues, thus enhancing the thermal effects of ultrasound. This theory has been refuted several times. In 2 studies, an ice pack was applied to human tissue for 5 minutes prior to ultrasound. The ice dropped the temperature so much that the ultrasound treatment could not raise the tissue temperature to even 50% of those who received identical ultrasound with no prior ice treatment.^14,15^ Ultimately, it does not make sense to cool something that you immediately want to heat, particularly if you have limited time with the patient and cannot allow the tissue to equilibrate.

## Myth 6. Tendons, ligaments, and muscle tissue heat at the same rate during thermal ultrasound treatments.

### Truth 6.

Tendons heat up 3 times faster than muscle tissue when 3MHz ultrasound is used.^16,17^ Why does this happen? Muscle tissue has more blood flow than tendons. Thus, the increased blood flow serves to prevent excessive heating since it can pull the heat away from the area of injury. However, since tendons have little blood flow, they heat up quicker. It is important to keep in mind that the dose and duration must be adjusted to prevent overheating of the tendons during the ultrasound treatment.

Non-portable in-clinic ultrasound is one of the most frequently applied therapeutic modalities in the world. It can be a very effective treatment, but does require patient access to the healthcare provider multiple times per week which can prove challenging to some patients – especially during the COVID-19 pandemic! In the past decade, a small, wearable ultrasound device has been developed for home use. The sam® (Sustained Acoustic Medicine) device is wearable and self-administered that provides up to 4 hours of treatment a day to treat injuries.

**Table:** 

SAM	Traditional ultrasound
**Treatment duration**	
Up to 4 hrs.	5–15 mins
**Frequency**	
3MHz	1.5MHz
**Indications**	
Soft tissue injuries, pain	Same
**Effective daily dose**	
18,720 Joules	~ 2,000–5000 Joules
**Heating**	
About half as much as traditional ultrasound	
**Intensity**	
0.132 W/cm^2^	0-5W/cm^2^
**Size of crystals**	
2 at 6cm^2^	2.5cm^2^
**Portable**	
Yes	No

The benefits of SAM over other non-portable ultrasound devices is that it is capable of delivering daily ultrasound at home or in the office for up to 4 hours at a time. A joule is the amount of energy per treatment. Traditional ultrasound is applied for 5–15 mins. If the intensity is high, between 2,000–5,000 Joules may be produced. However, the 4 hour sam® treatment can produce nearly 4 times as much (18,000 joules) as traditional ultrasound. The portable sam® device can be used at home at any time of the day and during any activity; however, traditional ultrasound requires application by a therapist in the clinical facility.

This review helped clarify several misconceptions about the administration of ultrasound. In addition, it provided a comparison of commonly used in-clinic use of therapeutic ultrasound versus newer wearable sam® technology. With appropriate parameters, one can find ways to use both in-clinic and wearable ultrasound to effectively treat injuries.^18,19^
